# Surface oxygen concentration on the Qinghai-Tibet Plateau (2017–2022)

**DOI:** 10.1038/s41597-023-02768-x

**Published:** 2023-12-15

**Authors:** Xiaokang Hu, Yanqiang Chen, Wenyixin Huo, Wei Jia, Heng Ma, Weidong Ma, Lu Jiang, Gangfeng Zhang, Yonggui Ma, Haiping Tang, Peijun Shi

**Affiliations:** 1 State Key Laboratory of Earth Surface Processes and Resource Ecology, Beijing Normal University, Beijing, 100875 China; 2 Faculty of Geographical Science, Beijing Normal University, Beijing, 100875 China; 3 Sichuan University, Chengdu, 610065 China; 4 Academy of Plateau Science and Sustainability, People’s Government of Qinghai Province and Beijing Normal University, Xining, 810016 China

**Keywords:** Environmental impact, Biogeochemistry

## Abstract

For the ecologically vulnerable Qinghai-Tibet Plateau (QTP), hypoxia is increasingly becoming an extremely important environmental risk factor that significantly affects the health of both humans and livestock in the plateau region, as well as hindering high-quality development. To focus on the problem of hypoxia, it is especially urgent to study the surface oxygen concentration (i.e., oxygen concentration). However, the existing research is not sufficient, and there is a lack of oxygen concentration data collected on the QTP. In this study, through the Second Tibetan Plateau Scientific Expedition and Research and field measurements, the oxygen concentration data and corresponding geographic environmental data were collected at 807 measurement points on the QTP from 2017 to 2022, and the spatiotemporal oxygen concentration patterns were estimated. This work filled the gaps in the measurement and research of oxygen concentrations on the QTP while providing data support for analyses of the influencing factors and spatiotemporal characteristics of oxygen concentrations, which is of great significance for promoting the construction of ecological civilization in the QTP region.

## Abstract (summary)

**Table:** 

**Measurement(s)**	Surface oxygen concentration
**Technology Type(s)**	Field measurement, model estimation
**Factor Type(s)**	Geographic location, raster
**Sample Characteristic - Environment**	Land cover
**Sample Characteristic - Location**	Qinghai-Tibet Plateau

## Background & Summary

The Qinghai-Tibet Plateau (QTP) is the “third pole” of the Earth^1^, with a total area of approximately 2.5 × 10^6^ km^2^ and an average elevation of over 4,000 m above sea level^2^. The air is thin, and the ecological environment is extremely vulnerable^3^. Hypoxia is a major environmental characteristic of the QTP^4^. Altitude sickness caused by hypoxia not only affect the daily life and health of short-term travelers but also have an impact on the life expectancy of permanent residents^5,6^. In recent years, with the regional development of the QTP and the completion and opening of the Qinghai-Tibet Railway, the permanent population in the region is increasing, and the short-term traveler population is growing rapidly^7,8^. From 2001 to 2020, the total population of Tibet and Qinghai increased from 7.87 million to 9.57 million, and the annual number of tourists increased from 4.48 million to 68.62 million^9,10^. Against the backdrop of high-quality regional development, hypoxia is increasingly becoming an extremely important environmental risk factor on the QTP. However, due to the underdeveloped health care system in the region, the increasing population exposure, and the low human adaptive capability to the hypoxic environment, the health risks of hypoxia for the population on the QTP are becoming increasingly serious^11^. Therefore, to obtain the surface oxygen concentration (i.e., oxygen concentration) data of the QTP and analyze the influencing factors are very important.

The oxygen concentration has been considered nearly constant since 1912, when Benedict proposed the composition of the atmosphere and the volumetric concentration of oxygen in the air^12^. Machta and Hughes collected observations of oxygen concentration between latitudes 50°N and 60°S, again indicating that the oxygen concentration in dry air was nearly constant at 20.946%^13^. Some studies have shown that atmospheric pressure and oxygen partial pressure decrease with increasing elevation, but the oxygen concentration does not significantly change at different elevations^14,15^. Through field research on the QTP and its surrounding areas^4,11,16^, we have discovered that oxygen concentration is not constant and exhibits spatiotemporal variations. Importantly, elevation is not the primary or sole factor controlling oxygen concentration; other factors such as temperature and vegetation also influence oxygen concentration. The relative contributions of elevation, temperature, and vegetation to oxygen concentration are −39.58%, 35.50%, and 24.92%, respectively^17,18^.

Considering the unique high-elevation hypoxic environment and the increasingly prominent health risks that are caused by hypoxia on the QTP, we conducted 14 field surveys in this region from 2017 to 2022. Through route-based scientific expeditions and fixed-point measurements, we obtained oxygen concentration data and corresponding geographic environmental data from 807 measurement points. In addition, we measured the fractional vegetation coverage (FVC) of 51 measurement points on the QTP using large surface coverage sampling quadrats (1 km × 1 km). Furthermore, based on the measured oxygen concentration data, we estimated the spatiotemporal patterns of oxygen concentration on the QTP. This work filled the gap in the research of oxygen concentration on the QTP, which provided data support for the analysis of influencing factors and spatiotemporal characteristics of oxygen concentration. This research played an important role in deepening the understanding of the environmental risks that the people and economic systems face in high-elevation areas, including the QTP, under the background of global climate change. This study is significant for ensuring ecological security, improving the health and well-being of residents/tourists and livestock, and promoting stable, prosperous, and high-quality development in high-elevation areas.

## Methods

### Field measurement methods

**Fig. 1:**
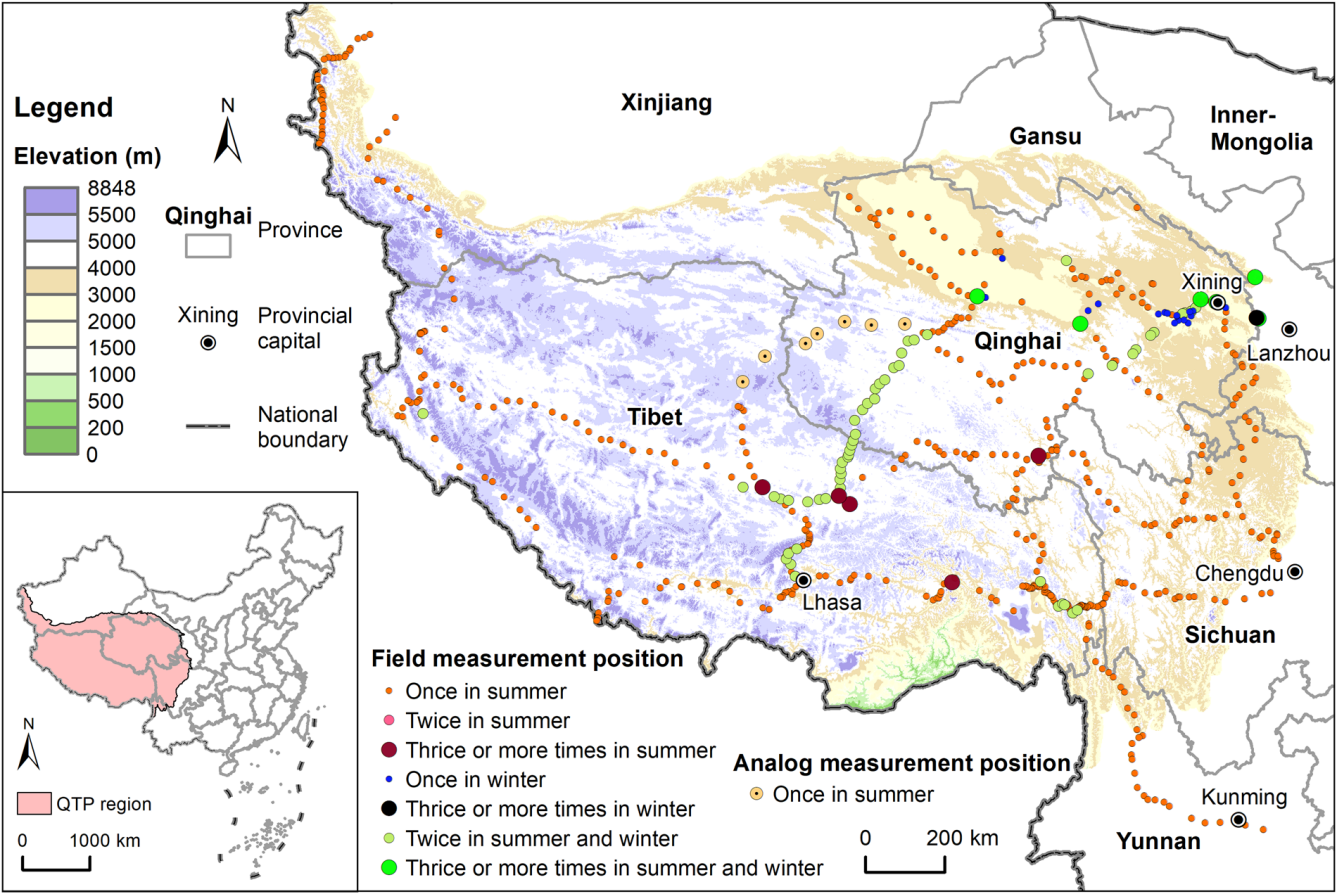
Oxygen concentration measurement position on the QTP from 2017 to 2022.

The QTP is vast, and its different regions exhibit distinct spatial heterogeneities in terms of the topography, landforms, climate, vegetation, soil, land use, and ecosystems. Considering that a limited number of measurement points are insufficient to represent the oxygen concentration variations in the entire region and would cause significant errors in the overall estimation, large-scale scientific field surveys are limited by factors such as manpower, resources, finances, and time. The layout of our field survey measurements was mainly based on two principles. First, spatially, given the tremendous spatial heterogeneity of the QTP and based on the actual traffic routes on the ground, we aimed to cover the different elevations as much as possible, including topography, landforms, climates, vegetation, soils, land uses, and ecosystem types on the plateau. Second, temporally, we concentrated the measurements during the vegetation growing season, which is typically from late July to early August each year, within a window of no more than one month to ensure the comparability of the data from different years, focusing the summer, while also considering autumn and winter. Based on this, we designed a “7 East‒West and 5 North‒South” scientific field survey route (Fig. 1) to conduct systematic observations of oxygen content and related geographic elements.

Following the overall layout of the scientific expedition route mentioned above, we conducted 14 route/point-based field surveys on the QTP from 2017 to 2022 (Table 1) and covering a total distance of over 30,000 kilometers. We obtained geographical environmental data, such as latitude and longitude, elevation, temperature, atmospheric pressure, relative humidity, and oxygen concentration, from 807 measurement points and used 1 km × 1 km field sampling quadrats to measure FVC at 51 measurement points on the QTP.

**Table 1:** Route/point oxygen concentration measurement from 2017 to 2022.

No.	Time	Field measurement route	Oxygen concentration measurement points	Vegetation coverage field quadrats
1	2017.07.27 - 08.04	Qushui-Lhasa-Nagqu-Golmud	65	0
2	2018.08.01 - 08.10	Lhasa-Shigatse-Nyalam-Saga-Ngari-Yecheng	67	9
3	2018.08.15 - 08.17	Around Qinghai Lake	13	2
4	2019.02.13 - 02.19	Around Qilian Mountains (winter)	53	0
5	2019.07.14 - 07.20	Around Qilian mountains (summer)	54	0
6	2019.07.27 - 08.04	Lhasa-Nyingchi-Ya’an-Chengdu	59	3
7	2020.06.22 - 06.29	Xining-Yushu-Qamdo-Kunming	75	8
8	2020.07.24 - 07.30	Yushu-Nagqu-Ngari-Zanda	61	8
9	2020.08.02 - 08.05	Xining-Hezuo-Hongyuan-Chengdu	40	3
10	2021.07.25 - 08.03	Yushu-Barkam-Maqin-Golmud-Mangya-Daqaidam-Xining	95	13
11	2021.10.22 - 11.19	Xining-Gonghe-Delhi-Golmud-Xining-Minhe	39	0
12	2022.07.15 - 07.25	Xining-Gonghe-Maduo-Qumarleb-Sonam Dargye Nature Conservation Station-Shuanghu-Nagqu-Lhasa	85	4
13	2022.07.17 - 07.23	Batang-Markam-Zuogong-Bangda-Basu	67	0
14	2022.07.27 - 07.30	Kashgar-Taxkorgan-Khunjerab-Kashgar	34	1
Total			807	51

At each measurement point, the latitude, longitude and elevation were recorded by GPS, and three groups of instruments (Table 2) were used to simultaneously measure the atmospheric pressure, temperature, relative humidity, and oxygen concentration approximately 1.5 meters above the ground^19^. To reduce the measurement error, the average of the data that were measured by the three groups of instruments was used as the final measurement result for each point. Since the oxygen concentration meter measures the instantaneous oxygen concentration in the quasi-stationary state of air, the data were measured in a windless environment as much as possible, and the data were read after the instrument was stabilized to minimize the effect of air flow on the measurement.

**Table 2:** Instruments used for measurement.

Data	Instrument	Time	Resolution
Longitude and latitude	Garmin Oregon 450 GPS / Garmin 63sc GPS	2017 / 2018-2022	1″
Elevation	Garmin Oregon 450 GPS / Garmin 63sc GPS	2017 / 2018–2022	1 m
Temperature / Relative humidity	DPH-103 digital temperature and humidity barometer	2018–2022	0.01 °C / 0.1%
Atmospheric pressure	Casio prg-130gc barometer	2017	5 hPa
	DPH-103 digital temperature and humidity barometer	2018–2022	0.1 hPa
Oxygen concentration	CY-12C digital oxygen concentration meter	2017	0.1%
	TD400-SH-O_2_ portable oxygen concentration meter	2018–2022	0.01%

It should be noted that due to the differences in the measuring instruments, the oxygen concentration data that were obtained in 2017 needed to be calibrated before comparison with data from other years. Additionally, the three TD400-SH-O_2_ portable oxygen concentration meters used for field measurements in 2018–2020 were purchased in 2018 and replaced in 2021 due to their life expectancy. Three new TD400-SH-O_2_ portable oxygen concentration meters were purchased in 2022. Therefore, the oxygen concentration data obtained in 2021 and 2022 also needed to be calibrated before comparison. For this reason, during the summer of 2022, we mutually calibrated the CY-12C and TD400-SH-O_2_ oxygen concentration meters that were used in 2017, 2018–2020, 2021, and 2022. We converted the oxygen concentration data that were obtained in 2017, 2021, and 2022 to equivalent data that were obtained in 2018–2020, which completed the calibration and unification of the data.

### Estimation methods

In the previous study, it can be found that elevation, temperature, and vegetation were the three most important factors that affected the oxygen concentration on the QTP^17,18^. Among these, elevation has a negative contribution to oxygen concentration, while temperature and LAI have positive contributions. Importantly, this relationship remains stable and does not vary with changes in time or space. Based on the location of the field measurement position from 2017 to 2021, the LAI in the corresponding locations and time periods were extracted^20^. Combining the measured elevation and temperature data, the relative contributions of the elevation, temperature, and LAI to oxygen concentration can be calculated as −39.58%, 35.50%, and 24.92%, respectively, by the correlation coefficient method and principal component analysis^18^. Since the data covered an extensive spatial range across the entire plateau and included measurements from both summer and winter seasons, it can be considered that the obtained relative contribution rates are highly relevant and applicable. Therefore, we assume that the effects and relative contributions of the major influencing factors of oxygen concentration on the QTP remain constant, allowing us to estimate the spatiotemporal distribution pattern of oxygen concentration.

**Fig. 2:**
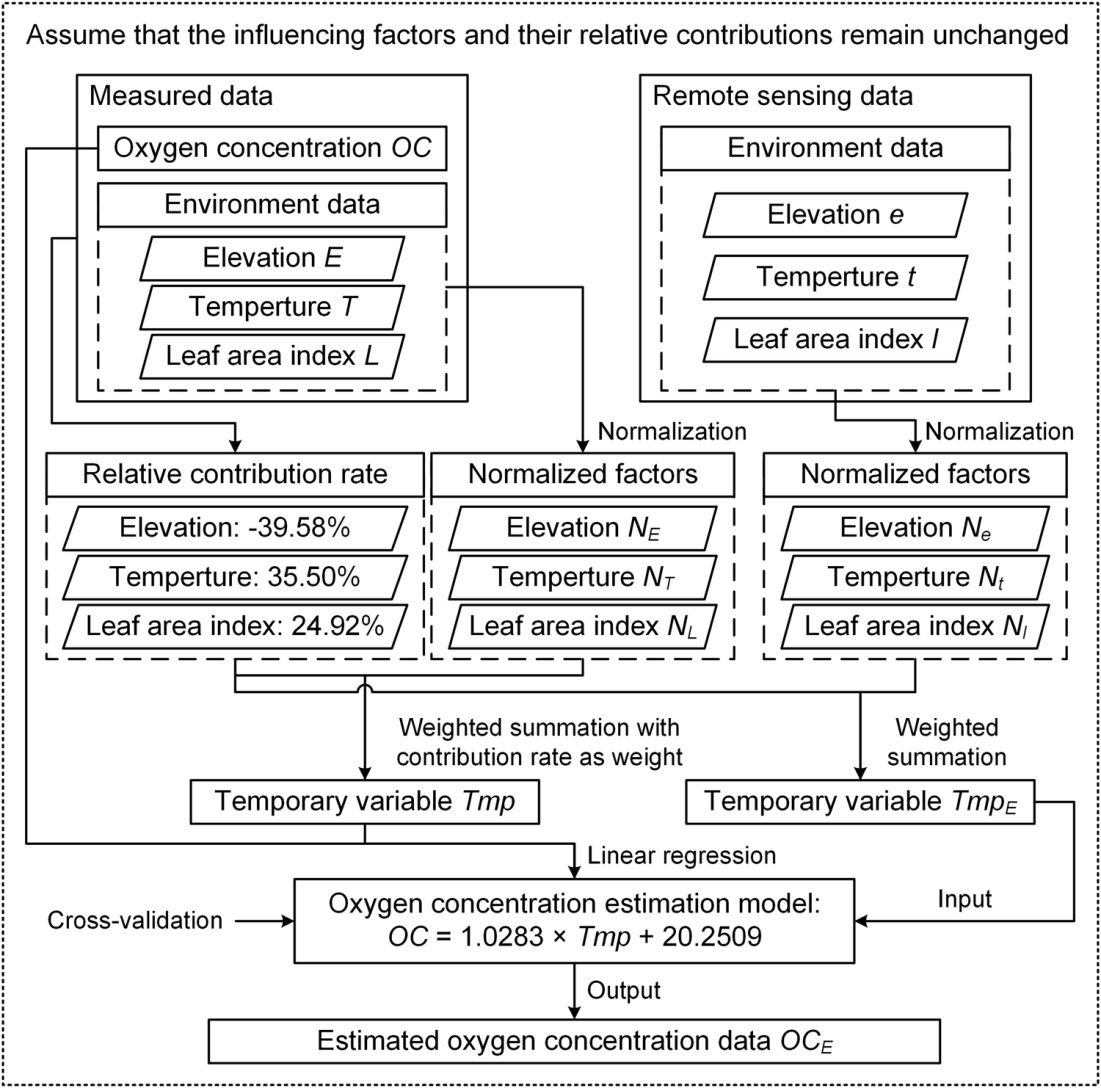
Calculation process of oxygen concentration estimation data on the QTP.

Considering the sample size and the stability of the data, the spatiotemporal patterns of oxygen concentration on the QTP were mainly estimated based on 422 groups of data that were obtained from field measurements on the QTP from 2018 to 2020^17^. The estimation process is shown in Fig. 2.

The measured elevation, temperature, and extracted LAI are standardized by using the Min-Max scaling method, resulting in normalized variables for elevation (*N*_*E*_), temperature (*N*_*T*_), and LAI (*N*_*L*_). These variables are then used as weights based on their relative contributions to oxygen concentration. Subsequently, a weighted sum is conducted to calculate the estimated intermediate variable (*Tmp*).

1 $$Tmp=-0.3958\times {N}_{E}+0.3550\times {N}_{T}+0.2492\times {N}_{L}$$

Based on the cross-validation method, we selected *m* groups of samples (*m* = 3, 4, 5, ⋯, 419) each time from the 369 groups of measured data as the training set to construct a linear regression model between the measured oxygen concentration and estimated temporary variable. The remaining (422-*m*) groups of samples were used as the testing set to validate the model. For each value of *m*, we performed 50,000 random trainings and used the average slope and intercept after training as the model parameters. We calculated the average and standard deviation of the root mean square error (RMSE) using the testing set. When the RMSE standard deviation was the smallest, the model was the most robust. Finally, we obtained the most robust linear regression model between the measured oxygen concentration (*OC*) and estimated temporary variable (*Tmp*), with a *R*^2^ of 0.72 and *p* < 0.001.

2 $$OC=1.0283\times Tmp+20.2509$$

The spatiotemporal estimation of oxygen concentration on the QTP was mainly based on DEM data^21^, monthly temperature data from 2001 to 2020^22^, and 8-day LAI data from 2001 to 2020^20^. The spatial resolution of the data was unified to 1 km × 1 km, and spatial clipping was performed according to the extent of the QTP. In addition, the temperature and LAI data were calculated as annual averages, as well as monthly averages for January and July.

Taking the estimation of the annual average oxygen concentration on the QTP as an example, the elevation, annual average temperature and annual average leaf area index were normalized, the relative contribution of each factor to the oxygen concentration was used as the weight, and the weighted sum was substituted into formula (1) grid by grid to obtain the raster data of the estimated temporary variable. Then, the estimated temporary variable data were substituted into formula (2) grid by grid to obtain the annual average oxygen concentration of the QTP. The estimations of average oxygen concentration in January and July were calculated in the same way. The difference between the July and January average oxygen concentrations was obtained by subtracting the average oxygen concentration in July from the average oxygen concentration in January.

## Data Records

The dataset “Surface oxygen concentration on the Qinghai-Tibet Plateau (2017–2022)” is available under National Tibetan Plateau Data Center^23^. A total of 5 data records are contained in the dataset. Of these,

1. Measured oxygen concentration data on the QTP (from 2017 to 2022) [File “Surface oxygen concentration on the Qinghai-Tibet Plateau (2017–2022).xlsx”];
2. Annual average oxygen concentration distribution data on the QTP [File “Annual average oxygen concentration.tif”];
3. January average oxygen concentration distribution data on the QTP [File “January average oxygen concentration.tif”];
4. July average oxygen concentration distribution data on the QTP [File “July average oxygen concentration.tif”];
5. Distribution data of the difference between the July average and January average [File “Difference between July and January.tif”].

### Measured data

The measured oxygen concentration data on the QTP were stored in a matrix with 13 columns and 807 rows. Among them, the 13 columns included the measured latitude, longitude, elevation, temperature, relative humidity, atmospheric pressure, oxygen concentration, FVC, and the geomorphologic and vegetation types were extracted from the Geomorphological Map of China (1:250,000)^24^ and the Vegetation Map of China (1:1,000,000)^25^, respectively. 807 rows represent the 807 groups of data we measured on the QTP from 2017 to 2022. As shown in Table 3, some of the measured oxygen concentrations on the QTP are shown with our measured points around Qinghai Lake from August 15th to August 17th, 2018, as an example.

**Table 3:** Oxygen concentration measured data on the QTP (partial).

TID	FID	Time	Longitude (°)	Latitude (°)	Elevation (m)	Temperature (°C)	Relative humidity (%)	Atmospheric pressure (hpa)	Oxygen concentration (%)	FVC (%)	Geomorphologic type	Vegetation type
18-068	QHL-001	2018/8/15 08:35	101.2273	36.6962	2659	16.21	73.3	742.8	20.35	/	Yellow River and Huangshui River valleys and basins small-region	Annual ripening food crops and hardy cash crop field, deciduous fruit tree orchard
18-069	QHL-002	2018/8/15 9:37	100.8533	36.9878	3209	14.54	75.1	701.7	20.28	/	Yellow River and Huangshui River valleys and basins small-region	Kobresia and forbs alpine meadow
18-070	QHL-003	2018/8/15 14:02	98.8713	37.1783	3831	17.41	54.9	643.1	20.30	92.20	South of Yellow River high mountains and basins small-region	Kobresia and forbs alpine meadow
18-071	QHL-004	2018/8/15 16:16	98.5498	37.0089	3118	20.58	46.7	703.7	20.43	/	South of Yellow River high mountains and basins small-region	Annual ripening food crops and hardy cash crop field, deciduous fruit tree orchard
18-072	QHL-005	2018/8/15 17:07	97.9563	37.0317	2936	16.41	51.9	718.0	20.36	/	Qaidam Basin small-region	Succulent saline dwarf subshrub desert
18-073	QHL-006	2018/8/15 18:17	97.3623	37.3730	2986	15.78	53.4	714.9	20.31	/	Qaidam Basin small-region	Subshrub and dwarf subshrub desert
18-074	QHL-007	2018/8/16 08:58	97.6494	36.9848	3061	13.27	70.8	709.7	20.38	/	Qaidam Basin small-region	Dwarf semi-arboreal desert
18-075	QHL-008	2018/8/16 10:04	98.0849	36.6686	3139	21.27	35.2	702.6	20.41	25.20	Qaidam Basin small-region	Subshrub and dwarf subshrub desert
18-076	QHL-009	2018/8/16 12:41	98.8741	36.7102	3229	18.37	42.0	696.1	20.52	/	Yellow River and Huangshui River valleys and basins small-region	Succulent saline dwarf subshrub desert
18-077	QHL-010	2018/8/16 14:54	99.6057	36.7544	3785	13.10	71.9	647.6	20.42	/	Yellow River and Huangshui River valleys and basins small-region	Kobresia and forbs alpine meadow
18-078	QHL-011	2018/8/16 16:30	99.8699	36.9810	3196	20.17	53.9	697.1	20.49	/	Yellow River and Huangshui River valleys and basins small-region	Temperate deciduous scrub
18-079	QHL-012	2018/8/16 17:02	99.9002	36.9787	3218	20.53	52.5	695.3	20.37	/	Yellow River and Huangshui River valleys and basins small-region	No vegetation
18-080	QHL-013	2018/8/17 17:00	101.7371	36.6396	2294	19.12	60.7	778.2	20.59	/	Yellow River and Huangshui River valleys and basins small-region	Annual ripening food crops and hardy cash crop field, deciduous fruit tree orchard

### Estimated data

**Fig. 3:**
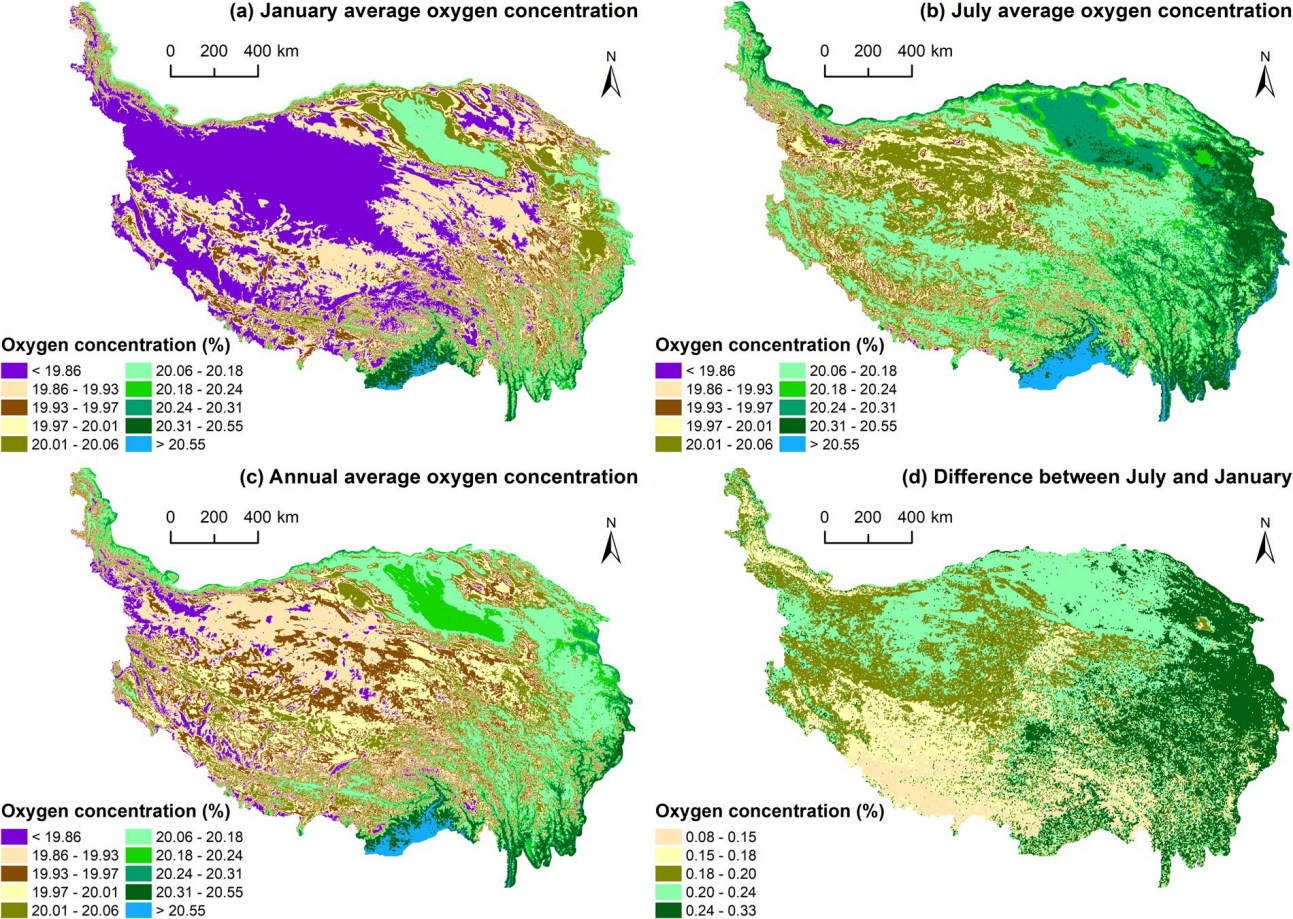
Estimated oxygen concentration distribution data on the QTP.

The estimated oxygen concentration distribution data (Fig. 3) on the QTP include the annual average, July average, January average oxygen concentration, and the difference between the July average and January average. The data were stored in GeoTiff format. The geographic coordinate system of the data was GCS WGS 1984. The spatial resolution of the data was 1 km × 1 km. The data had a spatial extent of 73.50°E to 104.67°E and 26.05°N to 39.91°N, with 2741 rows and 1663 columns, and the value of each pixel represented the oxygen concentration. The unit of the data was %.

From Fig. 3, temporally, the difference between July and January showed that the July average oxygen concentration on the QTP was higher than the January average, the July average oxygen concentration was higher than the annual average, and the January average oxygen concentration was lower than the annual average, which was related to the higher temperature and vegetation oxygen production in July. Spatially, the distribution of the annual, July, and January average oxygen concentrations on the QTP showed that the oxygen concentration first showed an obvious east‒west divergence pattern, i.e., the eastern part was higher than the western part, and the oxygen concentration decreased from east to west. Second, the oxygen concentration showed a pattern of north‒south belt alternation and extended east‒west. Third, the oxygen concentration showed a decreasing pattern from the periphery of the plateau to the hinterland as the elevation rose. The difference in oxygen concentrations between July and January showed a clear spatial pattern of east‒west and north‒south divergence, i.e., high in the northeast, low in the southwest, higher in the north and lower in the south.

## Technical Validation

### Potential error analysis

This study employed a route/point-based measurement approach using a portable electrochemical oxygen concentration meter. The instruments and their usage periods are detailed in Table 4. Specifically, the instruments used in 2017 were the CY-12C digital oxygen concentration meters, while the instruments used in 2018–2020, 2021, and 2022 were the TD400-SH-O_2_ portable oxygen concentration meters.

**Table 4:** Oxygen concentration meter used for measurement.

Time	Instrument	Working temperature	Working relative humidity	Range	Resolution
2017	CY-12C digital oxygen concentration meter	0 °C–40 °C	0%–90%	0.0%–50.0%	0.1%
2018–2020 2021 2022	TD400-SH-O_2_ portable oxygen concentration meter	−40 °C–70 °C	0%–95%	0.00%–30.00%	0.01%

#### Instrumental error

To mitigate instrumental errors, all instruments were calibrated with oxygen standard gases before each measurement. Three sets of instruments were simultaneously used for measurements, and the average data obtained from these measurements were taken as the results. However, it’s important to note that the instruments used in 2017 were the CY-12C digital oxygen concentration meters, which differ from the TD400-SH-O_2_ portable oxygen concentration used from 2018 to 2022. Furthermore, there were differences in the sensors of the instruments used in 2018–2020, 2021, and 2022. To address this instrumental error, we conducted field-based comparative measurements of oxygen concentration along the “Mado-Shuanghu-Bange” route using different instruments from July 15th to July 25th, 2022. For each measurement point, four groups of instruments were used simultaneously to take measurements (Table 5). Subsequently, the average of the data obtained from each group of instruments was taken during the data processing.

**Table 5:** Instrument information used in data calibration.

Group	Instrument	Number	Notes
A	CY-12C digital oxygen concentration meter	3	Previously used in 2017
B	TD400-SH-O_2_ portable oxygen concentration meter	3	Previously used in 2018 to 2020
C	TD400-SH-O_2_ portable oxygen concentration meter	3	Previously used in 2021
D	TD400-SH-O_2_ portable oxygen concentration meter	3	Newly purchased in 2022

**Fig. 4:**
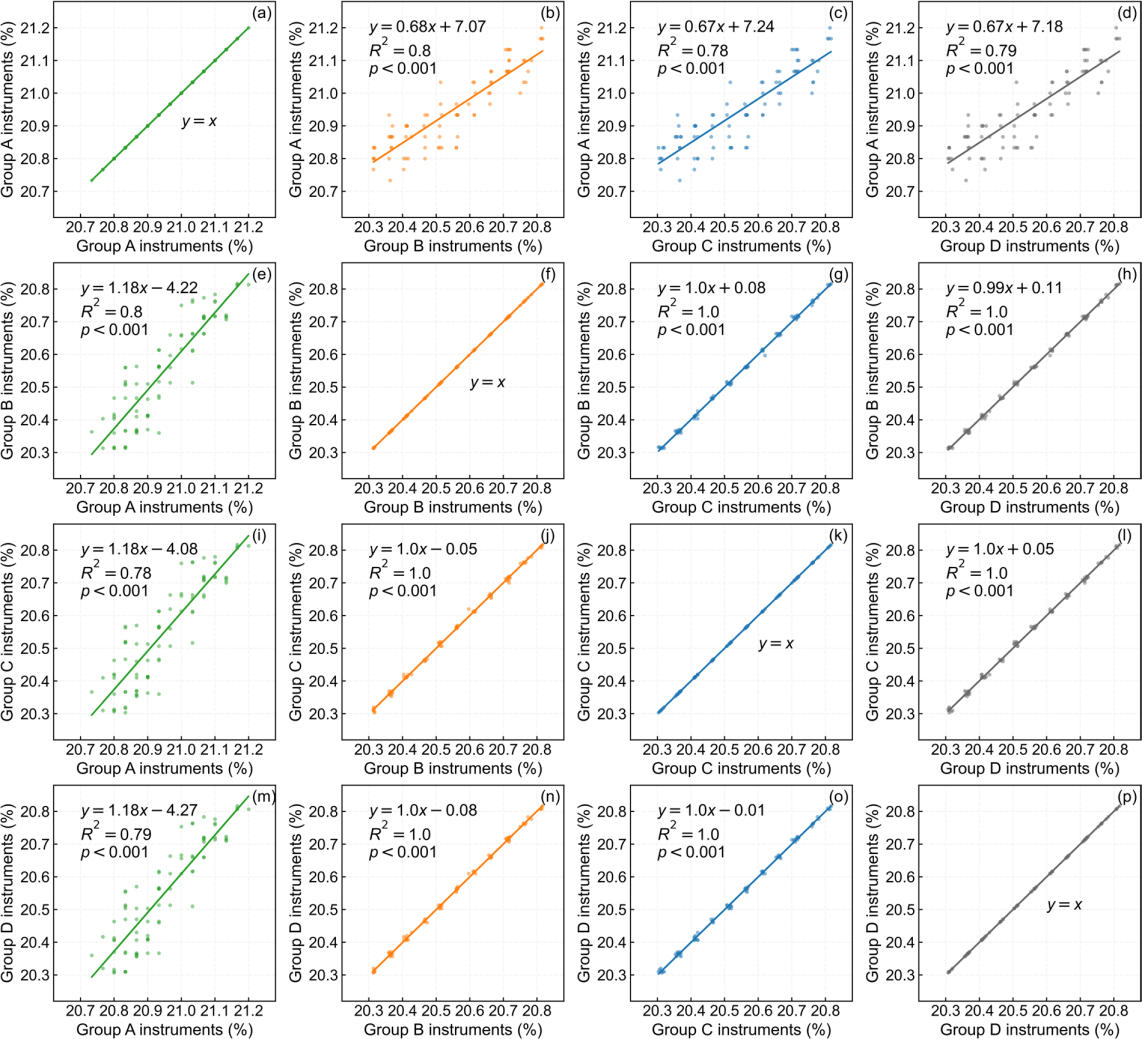
Relationship between the oxygen concentrations measured by different groups of instruments.

Since oxygen concentration was measured simultaneously, relationships were established by pairwise comparisons of data obtained from different groups of instruments (Fig. 4). Ultimately, the data were revised to adhere to the measurement standards of the same group of instruments, thereby eliminating instrumental errors.

The oxygen concentrations measured by the instruments in groups B, C and D showed excellent linear relationships with each other (Fig. 4), with *R²* values of 1.0, and all passed the significance test at the 0.001 level. The results indicated that there is almost no difference in the data measured by the TD400-SH-O_2_ portable oxygen concentration meters of groups B, C and D.

The data measured by the instruments in groups A and B (Fig. 4b,e), groups A and C (Fig. 4c,i), groups A and D (Fig. 4d,m) also showed good linear relationships with each other, with *R²* values above 0.7, and all passed the significance test at the 0.001 level. The results indicated that the difference in the data measured by the CY-12C digital oxygen concentration meters of group A and the TD400-SH-O_2_ portable oxygen concentration meters of groups B, C and D is small.

Considering the largest sample size of data measured in 2018–2020 (Table 1) and the best linear regression relationship between the data measured by instruments in group A and group B (*R*^*2*^ = 0.8). Based on the regression equation in Fig. 4, the oxygen concentration measured in 2017 (Fig. 4e), 2021 (Fig. 4g), and 2022 (Fig. 4h) was substituted into the equation as the independent variable, and finally the data were revised to the same standard to eliminate instrumental errors.

#### Environmental error

As indicated in Table 4, the CY-12C digital oxygen concentration meter operates in an environmental temperature range of 0 °C to 40 °C with a relative humidity range of 0% to 90%. On the other hand, the TD400-SH-O_2_ portable oxygen concentration meter operates in an environmental temperature range of −40 °C to 70 °C with a relative humidity range of 0% to 95%. We conducted field measurements during both summer and winter on the QTP, and during these measurements, we made every effort to ensure that the instruments operated in ideal environmental conditions to minimize the impact of the environmental factors such as temperature and water vapor on the measuring. The temperatures during the measurements ranged from −11.58 °C to 35.64 °C, and the relative humidity ranged from 7.9% to 93.2%, all of which met the requirements of the instruments (please note that temperature and relative humidity were not measured during the summer of 2017 due to the lack of instruments).

#### Methodological error

During measurements, the instruments were handheld to measure oxygen concentration at approximately 1.5 meters above the ground. Measurements of oxygen concentration were conducted in calm or low-wind environments whenever possible. It’s important to note that the fractionation effects can cause significant bias in oxygen concentration measurements when there is inconsistency in temperature, humidity, and pressure inside the instrument. Therefore, measurements were taken after ensuring as much stability and consistency as possible between the instrument’s internal and external environments to minimize errors.

#### Human error

Prior to each measurement, measurement personnel received training to ensure consistency in measurement methods. During measurements, the same person was responsible for reading the data to reduce errors caused by personnel changes.

### Measured oxygen concentration data validation

Considering that there were no previous oxygen concentration data on the QTP, it is generally believed in academia that the oxygen concentration is nearly constant at 20.946%^13^. Therefore, we conducted a one-sample t test of the 807 oxygen concentration data that were measured on the QTP during 2017–2022, with 20.946% as the comparison value. The statistical results (Table 6) showed that the oxygen concentrations that were measured in the summer and winter seasons of different routes were significantly different from 20.946%. This indicates to some extent that the oxygen concentration of the QTP is not constant and shows significant spatiotemporal differences with changes in geographical environment (elevation, temperature, and vegetation, etc.). At the same time, this also proves the relative accuracy of the measured data.

**Table 6:** Statistical characteristics of oxygen concentration on the Qinghai-Tibet Plateau.

Season	Year	Number of samples	Average	Standard deviation	Difference from 20.946%	
					Significance	95% confidence interval
Summer	2017	65	20.72	0.26	0.001	20.65–20.78
	2018	80	20.19	0.16	0.001	20.15–20.22
	2019	113	20.37	0.16	0.001	20.34–20.40
	2020	176	20.30	0.18	0.001	20.28–20.33
	2021	95	20.20	0.10	0.001	20.18–20.22
	2022	186	20.38	0.31	0.001	20.34–20.43
Winter	2019	53	20.16	0.11	0.001	20.13–20.19
	2021	39	20.01	0.12	0.001	19.97–20.05

### Measured FVC data validation

**Fig. 5:**
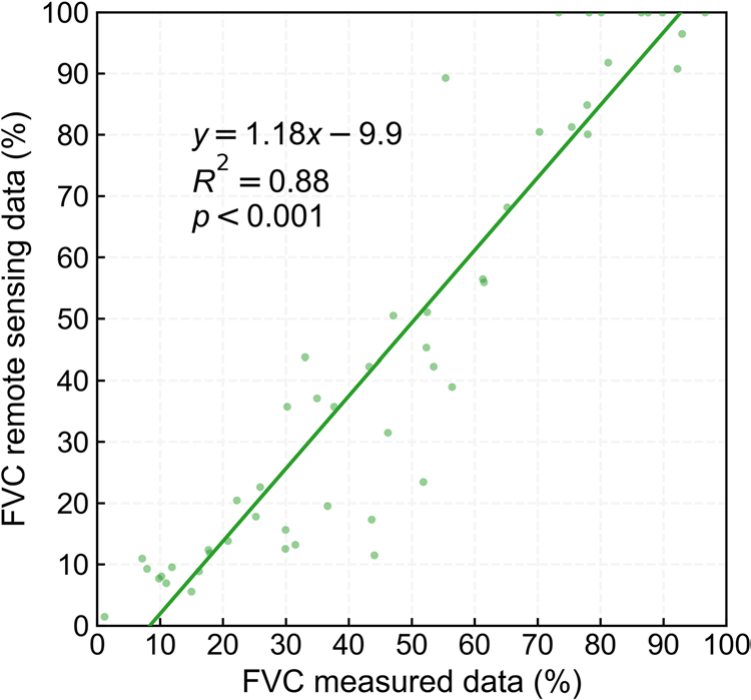
Measured FVC data validation.

During the field study on the QTP from 2018–2022, we measured the FVC of 51 measurement points by using 1 km × 1 km field sampling quadrats. Based on the China FVC dataset^26^, we extracted the FVC remote sensing data that corresponded to the measurement position and the corresponding month and established the relationship between the measured data and the remote sensing data to check the accuracy of the measured FVC. The result showed (Fig. 5) that the correspondence between the measured data and the remote sensing data was good, and the R^2^ of the linear regression model was 0.88 and passed the significance test of 0.001, which indicated that the measured FVC in the field was accurate and reliable. In addition, it also showed that using remote sensing data to obtain the FVC (or LAI) could be a reliable substitute for measured FVC in the oxygen concentration estimation.

### Uncertainty analysis of oxygen concentration estimation method

This study constructed the estimation model based on the cross-validation method. From the measured oxygen concentration data, a certain number of samples was selected as the training set to build the model each time, thus estimating the spatial distribution of oxygen concentration on the QTP. The standard deviation of oxygen concentration was calculated by statistically analyzing the oxygen concentration of each grid under all model estimations, which measures the uncertainty of the estimation method.

**Fig. 6:**
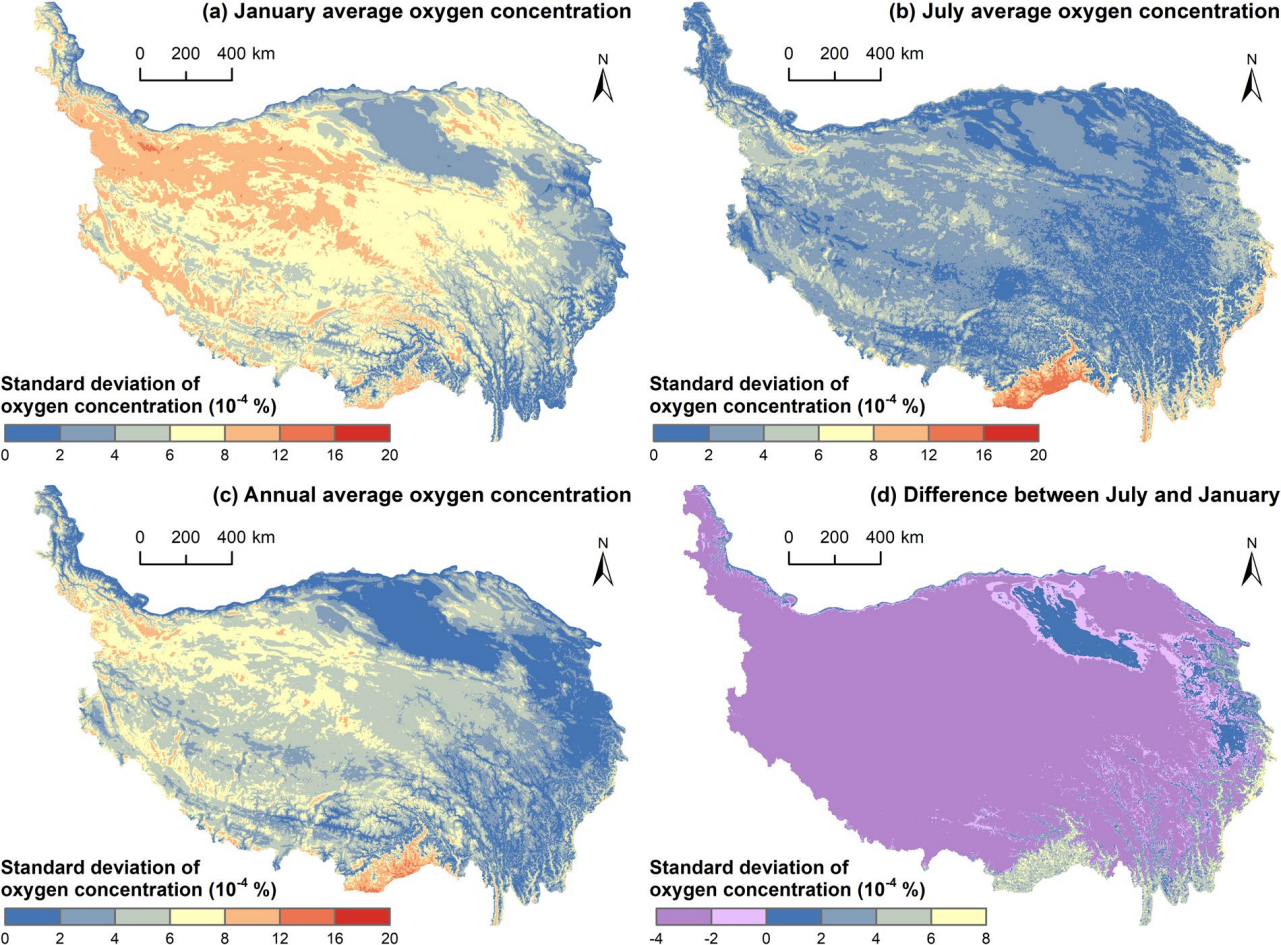
Uncertainty of the oxygen concentration estimation method.

From Fig. 6, the standard deviation of oxygen concentration is on the level of 0.0001%, indicating low uncertainty. Specifically, when estimating the January average oxygen concentration, the maximum standard deviation is 0.00192%, and the minimum is 0.00010%. When estimating the July average oxygen concentration, the maximum standard deviation is 0.00166%, and the minimum is 0.00010%. For the annual average oxygen concentration estimation, the maximum standard deviation is 0.00175%, and the minimum is 0.00010%. In most regions of the QTP, the standard deviation is less than 0.0012%. However, in the southern part of the QTP, the standard deviation is relatively large, ranging from 0.0012% to 0.0016%. This is mainly due to the significant elevation variations and spatial heterogeneity in this region, as well as the limited number of measurement points. Figure 6d shows that, except for areas such as the southern and eastern parts of the QTP and the Qaidam Basin, the uncertainty of the January average oxygen concentration estimation is higher than that in July. This is mainly due to the fact that among the data measured in the field from 2018 to 2020, the amount of data measured in summer was larger, with 369 groups, while the amount of data measured in winter was smaller, with only 53 groups. In summary, the oxygen concentration estimation method used exhibits low uncertainty and provides reasonably accurate estimations on the QTP.

### Estimated oxygen concentration data validation

By cross-validation, not only can a robust estimation model be established, but the model can also be validated. From the measured oxygen concentration data, a certain number of samples was selected each time as the training set to build the model, and the remaining samples were used as the test set to validate the model. For each training set number taken, 50,000 random trainings were performed, and the average and standard deviation of RMSE were calculated using the test set. Therefore, the relationship between the number of samples in the training set and the RMSE of the test set could be established.

**Fig. 7:**
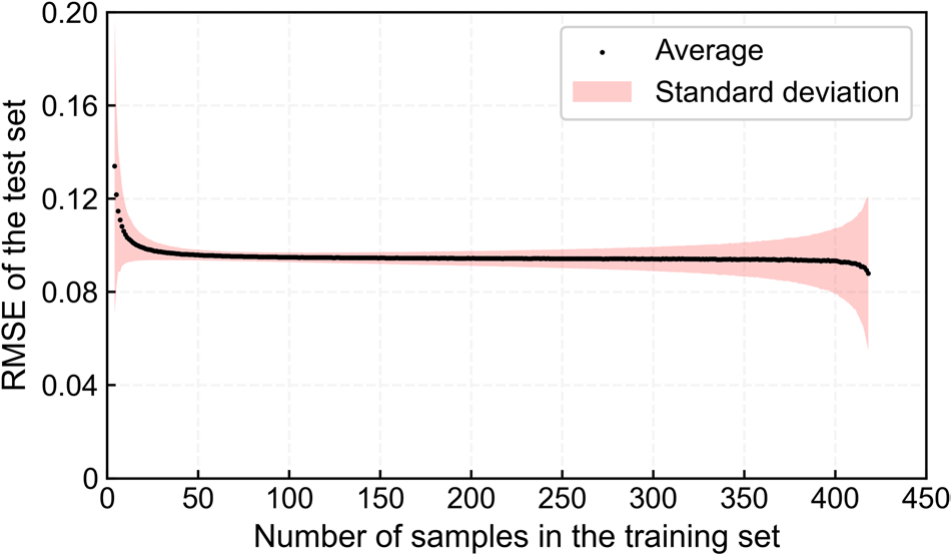
Oxygen concentration estimation model accuracy assessment.

From Fig. 7, as the number of samples in the training set increased, the corresponding averaged RMSE of the test set showed a downward trend. When the sample number of the training set was greater than 50, the averaged RMSE of the test set basically remained unchanged, slowly decreasing between 0.088–0.096. As the sample number of the training set further increased, the standard deviation of the RMSE of the test was larger due to the fewer samples in the test set. Considering both the average and standard deviation of the test set RMSE, the most robust estimation model of oxygen concentration on the QTP was constructed with a sample number of 76 for the training set (the standard deviation of the test set RMSE was the lowest, 0.0019), and the accuracy of the model was also relatively high (the average of the test set RMSE was 0.0952).

### Limitations and future work

Due to various factors, our data have certain limitations. (1) Our data were mainly obtained through route/point-based field surveys, and due to limitations in manpower, material resources, financial resources, and time, we could only measure the oxygen concentration on the QTP in a line-by-line manner and could not obtain large-scale and long-term oxygen concentration data. (2) During the measurement, we used portable electrochemical oxygen meters. Despite our efforts to minimize various errors, the precision of the oxygen concentration is limited due to the instrument’s resolution and measurement principles. (3) Due to the limited understanding of the factors that are affecting oxygen concentration, only the most important factors, such as the elevation, temperature and LAI, were considered when estimating the spatiotemporal patterns of oxygen concentration on the QTP, and the influence of factors such as wind and vegetation type were ignored. Additionally, our estimation of oxygen concentration distribution data was based on the assumption that the relative contribution rates of elevation, temperature and LAI to oxygen concentration remain constant. However, in reality, the relative contribution rates of these factors to oxygen concentration can vary in different regions and at different times. Due to the current limitations in data availability, it is challenging for us to calculate the specific relative contribution rates of each influencing factor to oxygen concentration in specific regions and at specific times. (4) We only considered the linear relationships between influencing factors and oxygen concentration. However, the impact of various factors on oxygen concentration may involve nonlinearity and complexity. The effects of these factors may not simply add up linearly, and there could be interactions among them.

To further observe the oxygen concentration on the QTP, we selected 64 meteorological stations in Qinghai, Tibet, Yunnan, Sichuan, Gansu and Xinjiang as observation points and established a network for observing the oxygen concentration on the QTP. This will enable long-term observation of oxygen concentration on the QTP and allow for a deeper analysis of the factors that influence oxygen concentration on the QTP based on the observation stations. In addition, we will further improve the estimation model of oxygen concentration on the QTP, fully consider the tremendous spatial heterogeneity, study the intrinsic relationship of the factors affecting oxygen concentration, and deeply analyze the spatial and temporal characteristics of the contribution of each factor to oxygen concentration, to more accurately estimate the oxygen concentration.

## Usage Notes

This dataset provides the measured and estimated oxygen concentration data on the QTP from 2017 to 2022, which is the first dataset of oxygen concentration on the QTP. This dataset fills the gap in the research on oxygen concentrations on the QTP and provides data support for oxygen concentration-related studies. For example, scholars can use these data to study the health risks of hypoxia in the people and livestock on the QTP^5^. Oxygen concentration is influenced by multiple factors, such as the elevation, temperature, and vegetation etc. A deeper understanding of the natural zones and geographic patterns of the QTP can be obtained based on the spatiotemporal distribution characteristics of oxygen concentration^27^.

At the same time, it should also be noted that due to the limitations in manpower, resources, finances, and time, the time span of the measured oxygen concentration data on the QTP is only from 2017 to 2022. The data records of oxygen concentration and related geographical environmental data only have 807 measurement points. Further, due to the limited accuracy of the model, the type of estimated data for oxygen concentration on the QTP included the annual average, January average, July average, and the difference between July and January, with a spatial resolution of 1 km × 1 km; the unit of the oxygen concentration data was %.

## Data Availability

The code in this study for constructing the oxygen concentration estimation model and estimating the oxygen concentration distribution data on the QTP were based on Python 3.9.2, and the key packages were *sklearn* and *osgeo*. The code can be found on GitHub (https://github.com/MysteriousBuddha/Surface-oxygen-concentration-on-the-Qinghai-Tibet-Plateau-2017-2022.git).
